# Cross-Species Transmission and Evolution of SIV Chimpanzee Progenitor Viruses Toward HIV-1 in Humanized Mice

**DOI:** 10.3389/fmicb.2020.01889

**Published:** 2020-08-11

**Authors:** Kimberly Schmitt, James Curlin, Leila Remling-Mulder, Ryan Moriarty, Kelly Goff, Shelby O’Connor, Mark Stenglein, Preston Marx, Ramesh Akkina

**Affiliations:** ^1^Department of Microbiology, Immunology, and Pathology, Colorado State University, Fort Collins, CO, United States; ^2^Department of Pathology and Laboratory Medicine, School of Medicine and Public Health, University of Wisconsin, Madison, WI, United States; ^3^Tulane National Primate Research Center, Tulane University, Covington, LA, United States; ^4^Department of Tropical Medicine, School of Public Health & Tropical Medicine, Tulane University, New Orleans, LA, United States

**Keywords:** HIV-1 evolution from SIVcpz, cross-species viral transmission, SIVcpz evolution into HIV-1, modeling SIV evolution into HIV using humanized mice, SIV pathogenesis in humanized mice, origin of human pathogens in NHP, SIV genetic changes toward HIV-1, viral adaptive changes and genetic evolution

## Abstract

The genetic evolution of HIV-1 from its progenitor virus SIV following cross-species transmission is not well understood. Here we simulated the SIVcpz initial transmission to humans using humanized mice and followed the viral evolution during serial passages lasting more than a year. All three SIVcpz progenitor viruses used, namely LB715 and MB897 (group M) as well as EK505 (group N) readily infected hu-mice resulting in chronic viremia. Viral loads increased progressively to higher set-points and the CD4^+^ T cell decline became more pronounced by the end of the second serial passage indicating viral adaptation and increased pathogenicity. Viral genomes sequenced at different time points revealed many non-synonymous variants not previously reported that occurred throughout the viral genome, including the *gag*, *pol*, *env*, and *nef* genes. These results shed light on the potential changes that the SIVcpz genome had undergone during the initial stages of human infection and subsequent spread.

## Introduction

Throughout history, cross-species transmission of animal viruses to humans gave rise to a number of deadly pathogens including HIV-1. Previous studies indicated that HIV-1 and HIV-2 likely arose from simian immunodeficiency viruses (SIVs) native to chimpanzees (SIVcpz), gorillas (SIVgor), and sooty mangabeys (SIVsm); respectively, into humans (Hirsch et al., 1989; Huet et al., 1990; Sharp et al., 1994; Chen et al., 1996; Hahn et al., 2000; Marx et al., 2001; Keele et al., 2006; Van Heuverswyn et al., 2006; Worobey et al., 2010; Sharp and Hahn, 2011). The chimpanzee-derived viruses are believed to have evolved into the most pathogenic HIV-1 group M viruses and to the less widespread HIV-1 group N, while the gorilla-derived viruses (SIVgor) are believed to have given rise to the rare HIV-1 group O (Keele et al., 2006; Van Heuverswyn et al., 2006; Sharp and Hahn, 2011; Hemelaar, 2012). Numerous other SIVs also exist in a wide range of non-human primates with no evidence of sustained transmission to humans despite frequent contact between these primates and humans (Worobey et al., 2010; Sharp and Hahn, 2011). While extensive analysis of HIV-1 and SIVcpz sequences cataloged the differences and similarities between these viruses, the exact mechanism by which the SIV genomes sequentially evolved into the current HIVs capable of efficient replication and causing disease remains to be elucidated. While humans and chimpanzees are highly related from an evolutionary and genetic standpoint, they do harbor very divergent and often unique host restriction factors that provide protection from various viral infections including those from NHP lentiviruses (Wain et al., 2007; Sauter and Kirchhoff, 2019). These factors, in combination with differences in host cell receptors such as CD4^+^ and chemokine receptors, such as CCR5 and CXCR4, pose considerable barriers that SIVs need to overcome to evolve into HIV-1 (Hvilsom et al., 2008; Locatelli and Peeters, 2012). A suitable model system needs to be employed to evaluate the critical questions surrounding the initial events of cross-species transmission and viral evolution during serial human propagation.

The new generation of humanized mice harboring a transplanted functional human immune system provides a suitable *in vivo* human surrogate system to address many questions in the context of SIV evolution (Akkina, 2013). Of the two common models that are currently used, the hu-HSC model is made by injecting human CD34^+^ hematopoietic stem cells (HSC) intrahepatically into the neonatal immunodeficient mice such as Rag2 DK mice, whereas the bone marrow/liver/thymus (BLT) mice are made by surgical transplantation of human fetal liver and thymic fragments under the murine kidney capsule (Garcia and Freitas, 2012; Shultz et al., 2012; Akkina, 2013). These mice produce T cells, B cells, macrophages and dendritic cells *de novo* on a continuous basis. The broad spectrum of these human immune cells at various stages of development makes the hu-mouse model particularly attractive to study human immune cell-tropic viruses (Berges et al., 2010; Denton and Garcia, 2011; Akkina, 2013).

Humanized mice have been used extensively for a host of HIV studies that encompass pathogenesis, sexual transmission and prevention, viral latency and testing of novel therapeutics to name a few (Neff et al., 2010; Denton and Garcia, 2011; Choudhary et al., 2012; Garcia and Freitas, 2012; Charlins et al., 2017; Hu et al., 2017). We and others have recently shown that these mice are permissive for SIV infections as well (Yuan et al., 2016; Schmitt et al., 2017, 2018; Sato et al., 2018; Yuan et al., 2018; Curlin et al., 2020). SIV propagation in hu-mice provides the selective environmental pressures necessary for the virus to potentially overcome human cell-imposed barriers potentially reflecting the original evolution of HIV-1 from its progenitors. Thus, many aspects of cross-species transmission by SIVs into the human host can be experimentally studied. In light of these advantages, recent studies, including ours, addressed the evolution of both HIV-1 and HIV-2 from their SIV ancestor viruses using the hu-mice system (Yuan et al., 2016; Schmitt et al., 2017, 2018; Sato et al., 2018; Yuan et al., 2018; Curlin et al., 2020). With regard to the HIV-2 progenitor virus, we have shown that SIVsm (SIVsmE041) native to sooty mangabeys was able to infect hu-mice and accumulate mutations that increase its fitness to human cells during sequential passages *in vivo* (Schmitt et al., 2017, 2018). Similarly, recent studies involving SIVcpz, the ancestor of HIV-1, showed that a number of SIVcpz strains can productively infect hu-mice resulting in viremia (Yuan et al., 2016, 2018; Sato et al., 2018; Curlin et al., 2020). Virus isolated after a single passage of 14–16 weeks duration harbored two notable mutations in the *env* gene (Yuan et al., 2016; Sato et al., 2018). However, these studies were limited in scope, since only portions of the viral genomes collected at a single time point approximately 14–16 weeks post-infection were analyzed. Additionally, repeated exposures necessary to mimic serial transmission to a new set of human hosts were not explored. Addressing some of these limitations, our recent preliminary report described the *in vivo* passage of strain SIVcpzEK505, which is closely related to HIV−1 group N (Curlin et al., 2020). Full genome sequence analysis was performed at multiple time points during infection to understand the dynamic nature of the genetic changes related to viral adaptation. Many sequence variants were noted throughout the genome, some of which became the predominant genotype within the viral population. Building on these observations, we undertook a comprehensive approach to evaluate the SIVcpz infection and evolution toward HIV-1 in hu-mice. We focused on four main aspects to improve upon previous studies. First, we sequentially passaged the SIVcpz viruses in hu-mice for much longer periods in 2 consecutive passages *in vivo* spanning more than a year. Three different strains of SIVcpz (MB897, LB715, and EK505), from groups M and N; respectively, were used. Second, viruses derived from the first passage were used to infect a second cohort of hu-mice to mirror serial transmission to a new human host. Third, we compared the viral loads and CD4^+^ T cell decline between the first and second passage viruses to determine potential increased viral fitness and pathogenicity over time. Finally, we derived sequence data on the entire genomes at different time points to identify the sequential genetic changes resulting from human selective pressure. Our results showed that longer passaging allowed the virus to accumulate many significant mutations not seen in previous reports, and there was increased viral fitness for growth in the human surrogate system accompanied by numerous mutations that increased pathogenicity.

## Results

### Chimpanzee Native Viral Strains SIVcpzEK505, LB715, and MB897 Can Establish Productive Infection and Chronic Viremia in Humanized Mice

**FIGURE 1:**
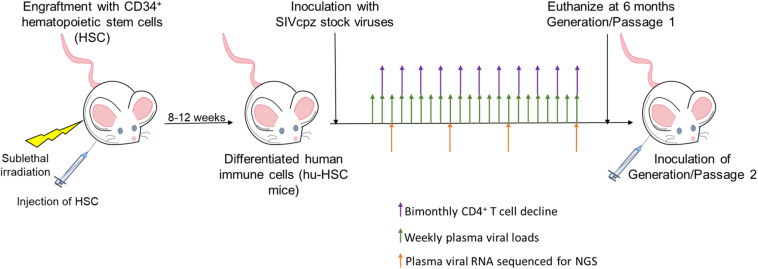
Viral passaging experimental scheme. Neonatal mice were sublethally irradiated and injected intrahepatically with human CD34^+^ hematopoietic stem cells as described in the Section “Materials and Methods.” Mice were screened 8–12 weeks post-reconstitution for human immune cell engraftment. Five hu-HSC mice with high human hematopoietic cell engraftment levels from a single cohort were infected intraperitoneally (i/p) with either cell-free SIVcpzEK505, LB715, or MB897 and monitored for plasma viral loads on a weekly basis using qRT-PCR. CD4^+^ T cell decline was assessed bimonthly by flow cytometry and plasma viral RNA was used for Illumina-based deep sequencing at 3, 11, 19-, and 24-weeks post-inoculation. The infected hu-HSC mice were euthanized at around 24 weeks and the isolated, infected cells were subsequently injected intraperitoneally into five naïve hu-HSC mice to begin the second passage/generation.

**FIGURE 2:**
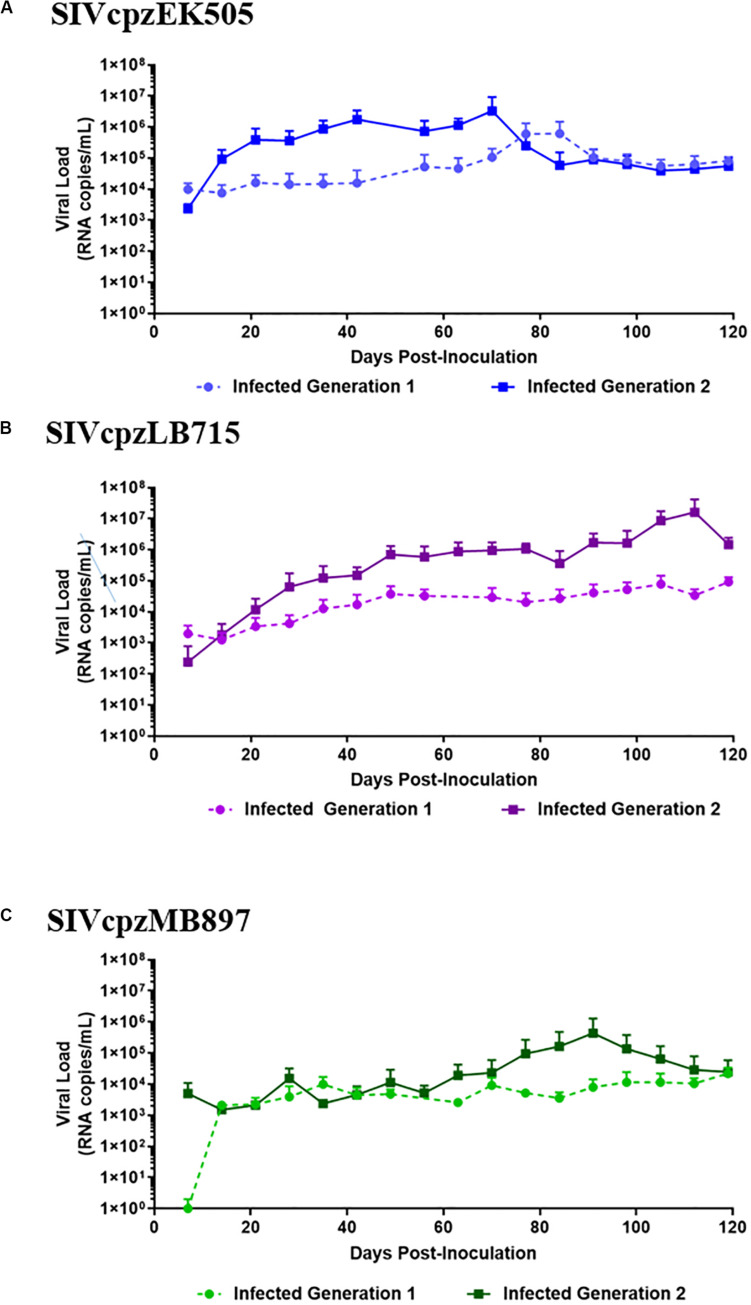
SIVcpz infection leads to detectable chronic viremia in humanized mice. **(A)** EK505, **(B)** LB715, and **(C)** MB897 first- and second-generation plasma viral loads. Five humanized mice were infected via i/p injections and the plasma viral loads were monitored by qRT-PCR on a weekly basis for the duration of the infection. No viral loads were detected in the uninfected control mice (data not shown).

To determine if SIVcpzEK505, MB897 and LB715 viruses of chimpanzee origin can establish productive infection, cohorts of five hu-HSC mice were inoculated with each of these and later serially passaged through different cohorts for a total of two generations (Figure 1). Plasma viral loads were assessed on a weekly basis using qRT-PCR with strain-specific primers and probes. Viral infection was evident within 14 days after the initial exposure with all the three SIVcpz progenitor strains demonstrating their ability to productively infect human cells *in vivo* (Figure 2). Plasma viral loads in SIVcpzEK505-infected hu-mice increased by 2-logs, peaking by 84 days post-inoculation. In comparison, viral loads peaked 14 days sooner during the second passage and were 1-log higher indicating enhanced replicative fitness of the virus (Figure 2A). With the SIVcpzLB715 viral strain, first passage viral loads increased slowly increasing through 161 days (>10^5^ RNA copies/mL) post-inoculation (data not shown). However, the viral loads became more robust during the second generation peaking at 2-logs higher (>10^7^ RNA copies/mL) and 35 days earlier (112 days post-inoculation) (Figure 2B). Thus, the SIVcpzLB715 virus also appears to have gained growth fitness in human immune cells over time. While still successful in initiating infection, viral loads with the SIVcpzMB897 were found to be lower than the other strains tested. Viral loads stagnated slightly above the limit of detection for most of the first passage period and peaked (≥10^4^ RNA copies/mL) at 119 days post-inoculation (Figure 2C). Unlike the other two strains above, the increase in second-generation plasma viral loads was slow for SIVcpzMB897 until around day 77. With the SIVcpzMB897 virus there was clearly a delay in viral adaptation to human immune cells during the first generation, and the viral growth appeared to evolve more rapidly during the middle of the second generation indicating eventual increased viral fitness. As expected, no plasma viral loads were detected in the control uninfected hu-HSC mice (data not shown). Previous data on HIV-1 BaL infections in hu-HSC mice showed that peak viral loads were reached at around 8 weeks post-inoculation (Berges et al., 2010). This is unlike the first generation of SIVcpz inoculations into hu-HSC mice where SIVcpzEK505, SIVcpzMB897 do not display peak viral loads till days 84 and 119, respectively, and SIVcpzLB715 does not peak during the first generation. In contrast, the viral loads peaked much earlier during the second generation, reflecting a greater adaptation toward human immune cells.

### Serial Passage of SIVcpz Viruses in Hu-Mice Leads to More Rapid CD4^+^ T Cell Decline

**FIGURE 3:**
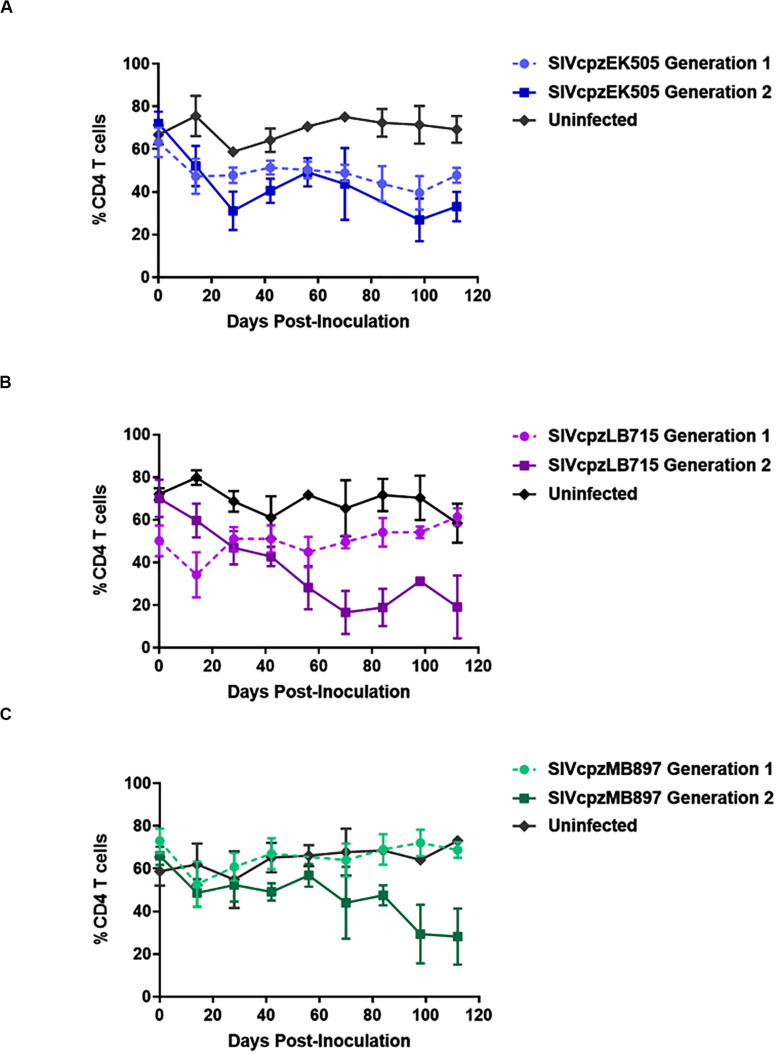
SIVcpz infection leads to a gradual decline in CD4^+^ T cells in humanized mice. The **(A)** EK505, **(B)** LB715, and **(C)** MB897 first- and second-generation percentage of circulating CD4^+^ T cells relative to the total CD45^+^/CD3^+^ cell populations were assessed on a bimonthly basis. Statistically significant depletion was seen in the second generation in infected hu-HSC mice relative to the uninfected control (two-tailed Student’s *t*-test, *p* < 0.001).

CD4^+^ T cell decline is a key aspect of HIV infection (Aldrovandi et al., 1993; Baenziger et al., 2006; Berges et al., 2006; Denton and Garcia, 2011; Akkina, 2013). Accordingly, we followed the CD4^+^ T cell levels in SIVcpz infected mice over time. The baseline human CD4^+^ T cell levels prior to infection in all mice was greater than 50% of all human CD3^+^ cells. By the end of second generation, CD4^+^ T cell decline became apparent in mice infected with any of the three viruses tested (*p* < 0.001), with varying levels of decline depending on the virus (Figure 3). The only SIVcpz strain with a noticeable decline in CD4^+^ T cells during the first passage was SIVcpzEK505. This decline was observed beginning at day 14 and was maintained over the course of 6 months (Figure 3A). However, it became slightly more pronounced during the second passage wherein decline was also seen within 14 days post-inoculation and persisted throughout the course of infection. SIVcpzLB715 and SIVcpzMB897 strains displayed no notable CD4^+^ T cell decline during the first passage, but SIVcpzLB715 infected mice showed rapid CD4^+^ T cell loss 14 days post-inoculation during the second passage (Figure 3B). While the SIVcpzMB897 second generation showed a much less precipitous drop in CD4^+^ T cells relative to the other SIVcpz strains, significant CD4^+^ T cell decline was still observed 98 days post-inoculation (Figure 3C). Overall, these results showing rapid decline of CD4^+^ T cells during the second passage versus the first suggest enhanced viral adaptation and pathogenicity. More importantly, this data demonstrates that all three progenitor SIVcpz viruses could establish productive viremia in hu-mice resulting in CD4^+^ T cell decline.

### SIVcpz Viruses Accumulate Adaptive Viral Genetic Changes During Long-Term Serial Passages in Hu-Mice

Selection pressures imposed by the human immune cell environment in hu-mice are likely to promote adaptive changes in SIV progenitor viruses. This “genetic bottleneck” arises from both physical and immunological conditions that prevent most variants within the founder viral population from establishing infection within the human host (Joseph et al., 2015; Kariuki et al., 2017). The adaptive changes to viral fitness may be mediated through changes that improve viral entry, replication, epistasis, immune escape, host restriction factors and/or pathogenicity. Furthermore, novel mutations in the virus may arise due to these host selection pressures on genes such as *vif*, *env*, or *nef* that counteract host restriction factors like TRIM5α, APOBEC3 proteins and tetherin. To assess the genomic changes occurring in SIVcpz viral strains during passages in humanized mice through two generations, we utilized Illumina-based deep sequencing to identify possible signatures of viral adaptation. This was performed on the genomes of SIVcpzEK505, LB715 and MB897 clonal stock viruses used for initial infections and the viruses sampled at roughly 3, 11, 19-, and 24-weeks post-inoculation during the first and second passages. The consensus sequence of each viral stock was used as a reference point for aligning the sequence reads of passaged viruses at each time point. Variants that occurred at a frequency of greater than 1% of the population relative to the reference sequence were identified and then characterized as either synonymous or non-synonymous. We looked for potentially adaptive non-synonymous variants based on several criteria including: (1) variants that arose during the first passage and increased in frequency amongst the viral population over time and (2) variants that appeared in multiple mice within a given passage across both passages. All three SIVcpz strains tested gave rise to many new non-synonymous variants that increased in frequency across both serial passages which were absent in the original founder stock viral sequences (Tables 1–3). Nine variants matching this criterion were identified in SIVcpzEK505 strain, seven in SIVcpzLB715, and with the greatest number of qualifying variants, 16, identified in SIVcpzMB897. All of these non-synonymous variants were absent in their respective stock virus population. The majority of substitutions occurring with at least 50% frequency at the last time point analyzed from the viruses sampled during the second passage were detected in the *env* gene, although other variants were found throughout the rest of the viral genome. SIVcpzMB897 had eleven non-synonymous changes in *env*, two within *gag* and *nef*, and one within *vif* meeting these criteria (Table 3). Based on their relative size, the *gag* and *pol* genes displayed very few substitutions when compared to the *env* gene, which is consistent with the previous knowledge that the *gag* and *pol* genes are highly conserved amongst retroviruses. Several of these substitutions identified that arose during the first passage were maintained at almost 100% frequency during the second passage in both replicates of mice from which the sequenced viral samples were obtained (Supplementary Figure 1). Relatively few substitutions were seen in *nef*, *vif*, *vpu*, and *vpr* genes in any of the SIVcpz strains tested which was surprising, given the role of these proteins in counteracting human restriction factors (Tables 1–3).

**TABLE 1:** Amino acid substitutions resulting from candidate adaptive mutations in SIVcpzEK505 passaged virus.

Protein	Position	Stock residue^*a*^	Variant residue^b^	P1 endpoint frequency^c^	P1 endpoint frequency^*d*^	P2 endpoint frequency^e^	P2 endpoint frequency^f^	SIV fraction^*g*^	HIV-1 fraction^h^
Gag	35	V	I	0.88	0.14	1.00	1.00	0.00	0.04
Vif	110	Y	N	0.00^i^	0.00^i^	0.72	0.30	0.00	0.00
Env	291	K	N	0.00^i^	0.00^i^	0.85	0.76	0.78	0.96
Env	402	V	G	0.56	0.00^i^	0.95	0.98	0.00	0.01
Env	414	P	S	0.00^i^	0.00^i^	0.90	0.64	0.00	0.04
Env	611	S	T	0.16	0.00^i^	0.81	0.94	0.61	0.95
Env	616	G	E	0.00^i^	0.00^i^	0.87	0.78	0.04	0.07
Env	668	R	K	0.30	0.00^i^	0.88	0.31	0.04	0.02
Env	703	E	K	0.60	0.39	1.00	0.99	0.00	0.00

**^*a*^Consensus amino acid from the stock virus sequencing dataset. ^*b*^Variant amino acid in passage 2 (P2) viruses. ^*c*^Frequency of the variant mutation in passage 1 (P1) replicate 1 sequencing datasets. ^*d*^Frequency of the variant mutation in passage 1 (P1) replicate 2 sequencing datasets. ^*e*^Frequency of the variant mutation in passage 2 (P2) replicate 1 sequencing datasets. ^*f*^Frequency of the variant mutation in passage 2 (P2) replicate 2 sequencing datasets. ^*g*^Fraction of SIV sequences from the HIV sequence compendium that contain the variant amino acid. ^*h*^Fraction of the HIV-1 sequences from the HIV sequence compendium that contain the variant amino acid. ^*i*^The 0 indicates below the limit of detection (1%) of our variant identification pipeline.**

**TABLE 2:** Amino acid substitutions resulting from candidate adaptive mutations in SIVcpzLB715 passaged virus.

Protein	Position	Stock residue^*a*^	Variant residue^b^	P1 endpoint frequency^c^	P1 endpoint frequency^*d*^	P2 endpoint frequency^e^	P2 endpoint frequency^f^	SIV fraction^*g*^	HIV-1 fraction^h^
Pol	329	E	K	0.00^i^	0.00^i^	0.84	0.55	0.00	0.01
Vpr	68	L	P	0.00^i^	0.00^i^	0.85	0.97	0.00	0.00
Vpu	45	R	T	0.00^i^	0.00^i^	0.66	0.79	0.00	0.00
Env	523	E	Q	0.00^i^	0.00^i^	0.50	0.76	0.00	0.00
Env	800	G	R	0.55	0.25	1.00	1.00	0.00	0.00
Env	841	G	R	0.00^i^	0.00^i^	0.47	0.60	0.00	0.00
Nef	36	D	N	0.00^i^	0.00^i^	0.83	0.93	0.00	0.00

**^*a*^Consensus amino acid from the stock virus sequencing dataset. ^*b*^Variant amino acid in passage 2 (P2) viruses. ^*c*^Frequency of the variant mutation in passage 1 (P1) replicate 1 sequencing datasets. ^*d*^Frequency of the variant mutation in passage 1 (P1) replicate 2 sequencing datasets. ^*e*^Frequency of the variant mutation in passage 2 (P2) replicate 1 sequencing datasets. ^*f*^Frequency of the variant mutation in passage 2 (P2) replicate 2 sequencing datasets. ^*g*^Fraction of SIV sequences from the HIV sequence compendium that contain the variant amino acid. ^*h*^Fraction of the HIV-1 sequences from the HIV sequence compendium that contain the variant amino acid. ^*i*^The 0 indicates below the limit of detection (1%) of our variant identification pipeline.**

**TABLE 3:** Amino acid substitutions resulting from candidate adaptive mutations in SIVcpzMB897 passaged virus.

Protein	Position	Stock residue^*a*^	Variant residue^b^	P1 endpoint frequency^c^	P1 endpoint frequency^*d*^	P2 endpoint frequency^e^	P2 endpoint frequency^f^	SIV fraction^*g*^	HIV-1 fraction^h^
Gag	35	V	I	0.00^i^	0.00^i^	0.99	0.64	0.00	0.04
Gag	105	E	K	0.00^i^	0.00^i^	0.90	0.74	0.69	0.02
Vif	47	E	K	0.88	1.00	1.00	1.00	0.00	0.01
Env	149	N	Y	0.54	0.68	0.97	0.75	0.00	0.01
Env	346	R	Q	0.00^i^	0.50	1.00	0.99	0.00	0.02
Env	351	S	N	0.00^i^	0.28	1.00	0.99	0.04	0.00
Env	413	G	R	0.83	0.64	0.99	0.99	0.00	0.00
Env	413	G	E	0.14	0.66	0.99	0.99	0.00	0.01
Env	414	K	R	0.33	0.00^i^	0.84	0.91	0.17	0.30
Env	442	N	I	0.00^i^	0.51	0.99	0.99	0.04	0.06
Env	475	L	V	0.08	0.60	0.74	0.52	0.87	0.50
Env	596	K	N	0.49	0.07	0.40	0.87	0.26	0.00
Env	702	F	L	0.17	0.10	0.90	0.61	0.04	0.29
Env	838	G	S	0.12	0.94	1.00	1.00	0.00	0.00
Nef	126	N	K	0.89	0.08	0.34	0.74	0.00	0.00
Nef	163	V	M	0.57	0.22	0.99	0.98	0.00	0.00

**^*a*^Consensus amino acid from the stock virus sequencing dataset. ^*b*^Variant amino acid in passage 2 (P2) viruses. ^*c*^Frequency of the variant mutation in passage 1 (P1) replicate 1 sequencing datasets. ^*d*^Frequency of the variant mutation in passage 1 (P1) replicate 2 sequencing datasets. ^*e*^Frequency of the variant mutation in passage 2 (P2) replicate 1 sequencing datasets. ^*f*^Frequency of the variant mutation in passage 2 (P2) replicate 2 sequencing datasets. ^*g*^Fraction of SIV sequences from the HIV sequence compendium that contain the variant amino acid. ^*h*^Fraction of the HIV-1 sequences from the HIV sequence compendium that contain the variant amino acid. ^*i*^The 0 indicates below the limit of detection (1%) of our variant identification pipeline*.*

Utilizing the sequence alignments in the HIV Sequence Compendium, we next sought to assess whether or not each non-synonymous variant observed produced a more “HIV-1-like” or “SIV-like” virus (Foley et al., 2017). We determined if a variant amino acid detected is present in a higher fraction of HIV-1 or SIV sequences (Tables 1–3). Two amino acid substitutions identified in Env for both SIVcpzMB897 (K414R and F702L) and SIVcpzEK505 (K291N and S611T) strains were found in a higher fraction of HIV-1 sequences than in those of SIVs’, indicating a change more representative of HIV than SIV. Interestingly, with the SIVcpzLB715 strain, the detected substitutions were neither more “SIV-like” nor “HIV-1-like” in nature. Additional passages *in vivo* of these SIVcpz viruses during a much longer time span may be necessary to observe the evolution toward HIV-1.

## Discussion

Here, we evaluated both the genetic and phenotypic changes that occurred during the evolution of progenitor SIV viruses toward HIV-1 using a humanized mouse model. In the first set of experiments, clonal stocks of three NHP SIV strains SIVcpz LB715, MB897 and EK505 belonging to groups M and N; respectively, were inoculated into hu-HSC mice to determine whether they can establish infection in the human surrogate host and affect CD4^+^ T cell levels. Later, the viruses were serially passaged in different cohorts of hu-mice to mimic serial human transmission and evaluate the potential increase in viral fitness and pathogenicity. While no animal model is perfect in fully mimicking the human system, the hu-HSC mouse model used here has several advantages over using human PBMC in *in vitro* experiments. These include, provision of a physiologic *in vivo* environment, immune pressures and prolonged time frame like 6 months or longer for serial viral propagation/adaptation. Each of the viral strains was able to infect hu-mice validating that SIVs originating from chimpanzees are capable of readily infecting human hosts as might have happened in the past. We followed the kinetics of viremia for several months in infected hu-mice and found there was a slow but steady increase in viremia over time (Figures 2A–C) during the first passage with each of the viral strains used. When the viruses from the first passage were used to infect new cohorts of hu-mice, viremia levels rapidly increased with the viral load set points being relatively higher than in the first passage. This showed that the SIVcpz strains were adapting to propagate better in the hu-mouse system as a consequence of increased fitness. Since helper CD4^+^ T cell decline is a hallmark of HIV-1 infection that results in immunosuppression, we also assessed this trait between the first and second passage of the hu-mouse adapted SIV strains. Whereas a slower pace of CD4^+^ T cell decline was noted during the first passage with all three viral strains over time, the cell decline became more pronounced and rapid during the second passage (Figures 3A–C). Both the increased amplitude of the viral loads and faster pace of CD4^+^ T cell decline seen with each strain during the second passage is indicative of viral evolution resulting in increased human pathogenicity.

While all the three SIVcpz viral strains tested above were capable of initiating infection in hu-mice, the SIVcpzMB897 strain displayed less robust viral loads than the others during the first passage. This contrasts with the conclusions of Sato et al. (2018) which reported that SIVcpzMB897 was more preadapted toward human immune cells compared to the other SIVcpz strains. Our findings are further supported by the longer timeframe required for noticeable CD4^+^ T cell decline by SIVcpzMB897 when compared to SIVcpzLB715 and SIVcpzEK505 strains. Furthermore, sequence data showed a greater number of variants in SIVcpzMB897 relative to the other two SIVcpz strains during the serial passage indicating it requires further adaptation for efficient propagation in humans.

**FIGURE 4:**
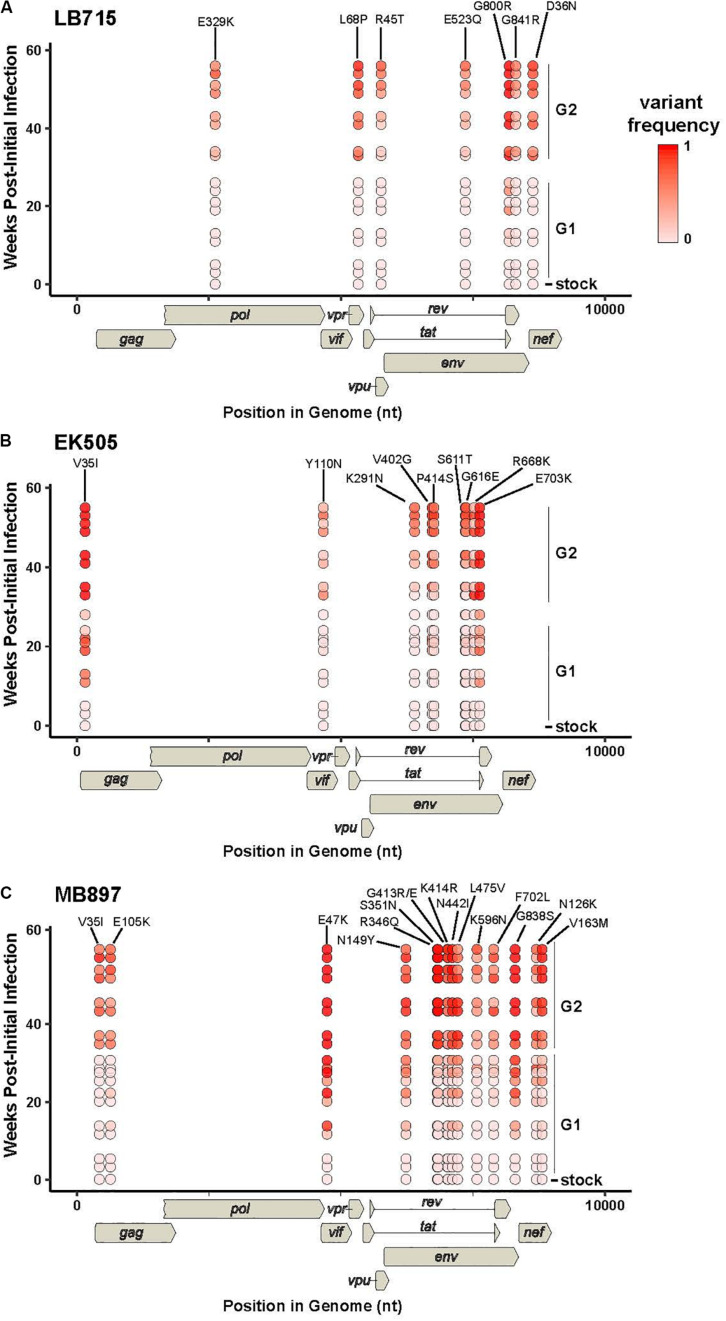
Viral variants increasing in population frequency from the first to the second generation. RNA samples from two mice per generation were sequenced. The frequencies of individual variants within a given timepoint sample from SIVcpz **(A)** LB715, **(B)** EK505, and **(C)** MB897 are plotted as a function of the weeks post-initial infection. Diagrams of the viral genomes are indicated below the *X*-axis. Non-synonymous variants that had a mean frequency of >0.5 in the final time point are shown. The viral variant frequency is indicated by the red color scale shown. Week 0 corresponds to the frequency in the viral stock pre-infection and the second generation was subsequently plotted beginning at week 30. For each time point, replicates are offset vertically from each other. The residue changes and numbers for each position are listed above their respective locations.

Based on sequence differences documented between SIVs and HIVs, it is clear that numerous genetic changes must have taken place in the progenitor SIV to give rise to the four known HIV-1 lineages (Keele et al., 2006; Van Heuverswyn et al., 2006; Sharp and Hahn, 2011; Hemelaar, 2012; Sauter and Kirchhoff, 2019). During this process, the progenitor SIVs had to evade or adjust to a number of native human cell barriers capable of interference with many steps in the viral life cycle. In addition to cell surface receptors involved in viral attachment, other barriers consist of restriction factors. These currently include, but are not limited to, TRIM5α, APOBEC3 proteins, SAMHD1, SERINC3/5, and tetherin which function by various mechanisms (Sheehy et al., 2002; Sayah et al., 2004; Stremlau et al., 2004; Neil et al., 2008; Le Tortorec and Neil, 2009; Berger et al., 2011; Goldstone et al., 2011; Serra-Moreno and Evans, 2012; McNatt et al., 2013; Etienne et al., 2015; Rosa et al., 2015; Usami et al., 2015; Ghimire et al., 2018; Meyerson et al., 2018). The list of restriction factors is continually expanding pointing to many other yet undiscovered intrinsic host defense mechanisms (Ghimire et al., 2018). These factors impose a strong selective pressure on non-native viruses to acquire adaptive changes. To identify potential genetic changes, we analyzed whole viral genomes from the first and second passages at different time points with a focus on non-synonymous genetic changes. Viral variants that increased in population frequency from the first to the second generation are presented in Figure 4. Some variants occurring in low frequency at the early time points became more prominent/dominant during later time points and became fixed (Tables 1–3). We also noticed several variants of transient nature that were seen at early time points which disappeared subsequently (data not shown).

Among the recent reports on SIV infection and evolution in humanized mice (Yuan et al., 2016, 2018; Sato et al., 2018), only limited regions of the viral genomes were evaluated by sequence analysis in one report and only variants from short-term infection (14–16 weeks of infection) at a single terminal time point were analyzed in all three studies (Yuan et al., 2016, 2018; Sato et al., 2018). To extend and improve upon these early studies and increase the breadth of analysis, here we examined the viral variants sampled at multiple time points during a much longer period spanning more than a year. Sequential passages performed in different cohorts of hu-mice also allowed the recapitulation of serial viral transmission and subsequent spread to a new human host. Not surprisingly, numerous changes could be identified throughout the viral genome beyond the two mutations described in the envelope gene in the studies mentioned above (Yuan et al., 2016; Sato et al., 2018).

Among the many non-synonymous changes seen, of note is the V35I substitution in Gag. This arose independently in both the EK505 (Group N) and MB897 (Group M) strains. While its exact function needs to be determined, that it arose in two divergent progenitor viral strains points to an essential role for it in human immune cell adaptation. However, this change was not seen in the LB715 strain (also group M, like MB897). More passages of LB715 may be needed for it to appear at a later time point or alternatively, other mutations in the vicinity might be playing a compensatory role. We also looked for the potential emergence of an important Gag M30K/R substitution, described previously in elegant studies by Wain et al. (2007), Sharp and Hahn (2011), and Bibollet-Ruche et al. (2012) detailing the origins of HIVs from NHP viruses. This mutation was shown to be highly conserved in HIV-1, but absent in most SIVs, suggesting it being a species-specific signature. However, it was not detected in our present studies. Corroborating our findings here, results from recent reports on SIVcpzMB897, SIVcpzEK505 and other chimpanzee-derived strains passaged in hu-mice also did not detect this Gag M30K/R substitution (Yuan et al., 2016; Sato et al., 2018). Thus, this substitution may not be as critical as previously thought for human cell adaptation, at least to overcome intracellular barriers during the early stages of human propagation. Supporting this hypothesis is the observation by Sato et al. (2018) that introduction of this substitution into SIVcpzMB897 strain did not augment viral fitness and replication kinetics in hu-mice. Alternatively, another variant the V35I substitution namely seen here may be playing an equivalent role similar to the Gag M30K/R substitution.

The viral envelope protein is involved in host cell receptor binding and dictates the efficiency of initial viral host cell interactions. Importantly, a non-synonymous change in Env identified in strain MB897 in two previous reports and found to improve viral pathogenicity, the Env G413R(/E) mutation, was also detected in our studies (Yuan et al., 2016; Sato et al., 2018). This variant appeared at 11 weeks post-inoculation and later became fixed. Its detection in three independent studies from different laboratories makes a compelling case for its critical nature in the SIVcpzMB897 strain’s adaptation to human cells. During the course of our two serial passages, we also observed two additional mutations of significance, namely, the Env N442I and K414R substitutions in the MB897 strain. These increased in frequency during the first passage and remained stable through the second passage. Env N442I is located adjacent to a well-characterized CD4 binding site in the V5 loop of envelope and potentially aids in facilitating viral attachment (Xiang et al., 2002; Chen et al., 2005; Decker et al., 2005; Yu et al., 2010). Another mutation, the Env K414R change, located in the CD4 binding site itself appeared later and began to increase in frequency during the second passage. It is possible that the initial adjacent mutations “prime” the site itself to better accommodate more drastic changes to the site, which may explain the delayed appearance of the Env K414R mutation relative to the others. The other two SIV strains tested also showed adaptive changes in receptor interacting sites. In SIVcpzEK505, an Env variant V402G in the CD4 binding site appeared during the late first passage (week 19) and eventually made up 100% of the viral population by the second passage. Another substitution directly adjacent to the chemokine binding site, the Env P414S, was detected at the beginning of the second passage (Xiang et al., 2002; Chen et al., 2005; Decker et al., 2005; Yu et al., 2010). These changes may have contributed to increased pathogenicity of SIVcpzEK505 as evidenced by both increased viral loads and more rapid CD4^+^ T cell decline that commenced at the beginning of the second passage. Similarly, the Env E523Q mutation seen in SIVcpzLB715 is located in the CD4 binding site and appeared during the second passage (Xiang et al., 2002; Chen et al., 2005; Decker et al., 2005; Yu et al., 2010). By evolving the CD4 and chemokine receptor binding sites, these viruses may be gaining improved engagement of the cell receptors and an overall higher fitness to the human host.

Several of the variants we detected did not appear until relatively late during the first hu-mouse passage (19–24 weeks post-infection) and did not become predominant until the second passage (Supplementary Figure 1). A number of other mutations were shown to arise at single time points and then disappear entirely indicating their transient nature and irrelevance in the context of viral evolution. These observations underscore the importance of longer *in vivo* viral passages and serial propagation to ascertain viral variants at multiple time points as they arise.

In addition to incompatible cell receptors barriers for viral entry, other major barriers for virus transmission is the myriad of host restriction factors such as TRIM5α, APOBEC3 proteins and tetherin. One of the functions of Vpu is to overcome tetherin. In some cases only a few genetic changes appear to be necessary to allow chimpanzee Vpu to be effective against human tetherin (Lim et al., 2010; Vigan and Neil, 2010; Kluge et al., 2013; Sauter and Kirchhoff, 2019). While we have not yet detected any variants directly connected to the known human restriction factors at these serial passage levels, several other significant variants with increased frequency are noted as shown in Tables 1–3, including the Vpu R45T substitution identified in SIVcpzLB715. We also identified two amino acid substitutions in both SIVcpzEK505 Y110N and SIVcpzMB897 E47K, which appeared fixed by the end of the second generation. Both of these variants were below the limit of detection in the starting stock viruses (Tables 1, 3). Additional Vif variants may continue to arise over repeated passaging in our humanized mouse model to further confirm candidate adaptive mutations that may contribute to counteracting the action of APOBEC3G (or APOBEC3 proteins) in human immune cells. These mutations and others need to be further characterized to determine their role in viral adaptation, pathogenesis and fitness. These mutation and others need to be further analyzed to determine their role in viral adaptation, pathogenesis and fitness. Two variants in Pol and Vpr, Pol E329K and Vpr L68P, that were identified in viral strain SIVcpzLB715 arose at the beginning of the second passage and then were maintained in over half of the viral population indicating their potential role for viral adaptation (Table 2). The Pol E329K mutation is located in the p51 reverse transcriptase subunit of the viral polymerase (Nagata et al., 2017) and the Vpr L68P substitution is found within the “LxxLL” motif (Kino et al., 1999; Kamata and Aida, 2000; Sherman et al., 2000; Kogan and Rappaport, 2011; Gonzalez, 2017). While, the function of the Pol E329K substitution is still unknown, the “LxxLL” motif is required for glucocorticoid-dependent activation of transcription to maintain resting and stress-related homeostasis as well as being directly implicated in the nuclear localization of the PIC complex (Kino et al., 1999; Kamata and Aida, 2000; Sherman et al., 2000; Gonzalez, 2017). This same mutation (Vpr L68P) was also identified as a proline (LxxLP) in HIV-1 NL4-3. This proline change has been noted in a participant sample but was not tested experimentally to confirm its effect on viral pathogenicity. However, previous studies have found that direct mutation of this residue leads to rapid progression of AIDS (Sherman et al., 2000; Gonzalez, 2017). Additionally, it is possible that some of these mutations may confer no additional fitness, but rather become fixed stochastically. However, the persistence of these mutations across multiple generations and immune environments make it unlikely that non-favorable mutations would increase in frequency within the viral population overtime. Further studies and passages of these viruses are needed to determine the direct function of these amino acid substitutions.

An important question is whether the non-synonymous variants seen here during serial passages in a human surrogate system reflect evolution toward HIV-1 as a whole or singly align with those of existing variants in the SIV genomes. Sequence alignments were done using the HIV Sequence Compendium (Foley et al., 2017). Specifically, we assessed whether the observed amino acid variants are present in a higher fraction of HIV-1 sequences than in SIV sequences. Most of the mutations we identified were present in very few SIV or HIV strains. However, two variants, Env K291N and Env S611T observed in the EK505 progenitor strain were found approximately 20% more frequently in HIV sequences than in SIV (Foley et al., 2017). However, these substitutions have no known function. The variant Env F702L seen in MB897 strain is located in the gp41 of Env adjacent to the Kennedy Epitope but no function is known (Steckbeck et al., 2011). None of the variants seen in the LB715 strain appeared to be common in either SIV or HIV strains. Several other variants seen were found be to more common in SIVs than in HIVs though the functionality of these changes remains unclear.

In summary, our results showed that HIV progenitor SIVcpz viral strains, from the pandemic Group M (MB897 and LB715) and non-pandemic Group N (EK505), can readily infect humanized mice leading to chronic viremia and upon further passage, marked CD4^+^ T cell decline. These findings reinforce the utility of the hu-mouse human surrogate system in modeling cross-species transmission and viral adaptation. Whereas the non-synonymous sequence changes seen in the CD4 and chemokine receptor binding Env regions of these evolving viruses show one aspect of enhancing viral affinity and fitness to the human host, other notable changes might contribute to other steps in viral replication. While the sequences of the variant viruses at the second passage spanning several months do not appear to be more like HIV, the SIV progenitors have responded to the selection pressure imposed by the *in vivo* human system by acquiring sequence changes. It remains to be seen if additional passages in hu-mice would impose further selection giving rise to viral strains more like HIV-1. Nevertheless, the ease with which these viruses infected humanized mice is remarkable, suggesting that many SIV strains currently circulating in the wild continue to pose a significant zoonosis risk to the human and have the potential to generate dangerous new HIV strains in the future. With the knowledge gained on how SIVcpz first adapted to human immune cells and gained virulence we will be able to better predict if, and how, other NHP derived lentiviruses may cross over into humans in the future.

## Materials and Methods

### Preparation of the SIVcpz Viral Stocks

In order to generate virus, full-length infectious molecular clones of pSIVcpzMB897, pSIVcpzLB715, and pSIVcpzEK505 were transfected into 293T cells. Briefly, 15 μg of plasmid DNA and 30 μl of TurboFect transfection reagent (Thermo Fisher Scientific, Waltham, MA, United States) was diluted in serum-free DMEM to transfect 293T cells seeded in a T150 flask. At 48 h, virus supernatants were harvested and clarified by low speed centrifugation. To concentrate the viral inoculum, clarified supernatant was ultracentrifuged on a 20% sucrose (w/v) cushion in Ultra-Clear tubes (Beckman Coulter, Pasadena, CA, United States). Ultracentrifugation was conducted utilizing a L8-70M ultracentrifuge and SW 28 Ti rotor (Beckman Coulter, Pasadena, CA, United States) at 27,000 rpm for 2 h at 4°C. Supernatant was decanted and pelleted virus was resuspended gradually on ice for 20 min in serum-free DMEM. Resuspended, cell-free virus was used to directly infect humanized mice, as described below, and an aliquot was used for titration on TZM-bl cells. Briefly, virus was serially diluted from 10^–1^ to 10^–6^ and used to inoculate TZM-bl reporter cells. At 48 h, media was removed, cells were washed with PBS and the monolayer was fixed using 1% formaldehyde-0.2% glutaraldehyde in PBS for 5 min. Cells were washed and incubated in a solution of 4 mM potassium ferrocyanide, 4 mM potassium ferricyanide, 4 mM magnesium chloride and 0.4 mg *X*-gal per ml, for 2 h at 37°C. The reaction was stopped and the tissue culture infectious dose (TCID_50_) per ml was calculated to determine virus titer (Derdeyn et al., 2000; Wei et al., 2002).

### Generation of Humanized Mice

Humanized mice were prepared using fetal liver-derived CD34 cells that were isolated, column purified (Miltenyi Biotec, San Diego, CA, United States), cultured and assessed for purity utilizing flow cytometry as previously described (Akkina et al., 1994; Bai et al., 2000; Veselinovic et al., 2016; Schmitt et al., 2017). Preconditioned by irradiation with 350 rads, neonatal Balb/c Rag1^–/–^γc^–/–^ or Balb/c Rag2^–/–^γc^–/–^ mice were intrahepatically injected with 0.5–1 × 10^6^ human CD34^+^ cells per mouse (Berges et al., 2008; Veselinovic et al., 2016). Human cell engraftment was then determined by flow cytometry in transplanted mice at 10–12 weeks post-reconstitution by collecting peripheral blood. The red blood cells were lysed using the Whole Blood Erythrocyte Lysing Kit according to the manufacturer’s instructions (R&D Systems, Minneapolis, MN, United States). Fractioned white blood cells were stained with mouse anti-human CD45 FITC (eBioscience), CD3 PE (eBioscience) and CD4 PE/Cy5 (eBioscience) for flow cytometry in order to confirm human cell engraftment (Berges et al., 2006; Veselinovic et al., 2016; Schmitt et al., 2017). All mice were maintained at the Colorado State University Painter Animal Center. The studies conducted in this publication have been reviewed and approved by the CSU Institutional Animal Care and Use Committee.

### SIVcpz Infection of Humanized Mice and Viral Load Determination by qRT-PCR

Mice with high (>60% CD45^+^, >50% CD4^+^) human hematopoietic cell engraftment levels were used. At 16 weeks post-engraftment, 200 μl of cell-free SIVcpzMB897 (2 × 10^5^ TCID_50_), SIVcpzEK505 (3.2 × 10^5^ TCID_50_), and SIVcpzLB715 (2 × 10^5^ TCID_50_) were used to inject five hu-HSC mice intraperitoneally (i/p). To assess plasma viral loads, peripheral blood was collected weekly by tail vein puncture using non-heparinized capillary tubes and transferred immediately to EDTA-containing vacutainer tubes (BD Biosciences, San Jose, CA, United States). The peripheral blood was mixed with PBS for a final volume of 150 μl and centrifuged for 5 min at 400 × *g*. Plasma was collected, and viral RNA was extracted from the plasma using the E.Z.N.A. Viral RNA kit according to the manufacturer’s instructions (OMEGA Bio-Tek, Norcross, GA, United States). Viral loads were determined using the iScript One-Step RT-PCR kit with SYBR green and the manufacturer’s instructions (Bio-Rad, Hercules, CA, United States). Virus-specific primers were designed based on a conserved region in the LTR of SIVcpzEK505, SIVcpzMB897 and SIVcpzLB715 (GenBank accession numbers: DQ373065, EF535994.1, and KP861923.1, respectively). The primers designed for qRT-PCR were as follows: (1) SIVcpzEK505: forward 5′-TAGTGTGTGCCCATCCATTCG-3′ and reverse 5′-CACCGCCAGTCAAAATTGCG-3′, (2) SIVcpzMB897 forward (5′-CCTCAGATATTAAGTGTCTGTGCGG-3′) and reverse (5′-GCTAGTCAAAAATTAGGCGTACTCACC-3′), and (3) SIVLB715 forward (5′-TGCTCGGACTCTGGTAACTA-3′) and reverse (5′-CCGCTACTTCTGGTTTCACTTTCACTT-3′). All primer sets listed above were used in a qRT-PCR reaction with the following cycling conditions: 50°C for 10 min, 95°C for 5 min, followed by 40 cycles of 95°C for 15 s and 60°C for 30 s in the Bio-Rad C1000 Thermo Cycler with the CFX96 Real-Time System (Bio-Rad, Hercules, CA, United States). The standard curve was prepared using a series of 10-fold dilutions of viral SIVcpzEK505, SIVcpzMB897, or SIVcpzLB715 LTR at a known concentration. The sensitivity of this assay was 1,000 copies per ml (1 copy per μl).

### Determination of CD4^+^ T Cell Levels

Peripheral blood was collected bi-monthly from infected and control mice by tail vein puncture. Human cell engraftment levels were assessed by flow cytometry. 5 μl of FcγR-block (Jackson ImmunoResearch Laboratories, Inc., West Grove, PA, United States) was added to the blood for 5 min. The blood was then stained with fluorophore conjugated hCD45-FITC, hCD3-PE and hCD4-PE/Cy5 (BD Pharmingen, San Jose, CA, United States) for 30 min. Erythrocytes were lysed using the Whole Blood Erythrocyte Lysing kit according to the manufacturer’s instructions (R&D Systems, Minneapolis, MN, United States). The stained cells were then fixed in 1% paraformaldehyde and 0.45 μm-filtered. To assess CD4^+^ T cell depletion in uninfected and infected mice, the CD3^+^ T cells levels were calculated as a ratio of the entire CD45^+^ (lymphocyte common antigen) population. The CD4^+^ T cell population levels were then determined as a percentage of the entire CD3^+^ T cell population. Baseline levels of the CD45^+^, CD3^+^ and CD4^+^ cells were measured prior to infection as a control. All flow cytometry data was analyzed using the FlowJo v10.0.7 software package (FlowJo LLC, Ashland, OR, United States). CD4^+^ T cell decline was assessed utilizing a two-tailed Student’s *t*-test (*p* < 0.001) to compare the two groups of mice, infected and uninfected.

### Illumina-Based Deep Sequencing and Analysis

For two mice of each generation, viral RNA from the plasma collected approximately at weeks 3, 11, 19, and 24 post-infection from two mice from each passage were used to synthesize the cDNA using SuperScript IV and the manufacturer’s instructions (Invitrogen, Carlsbad, CA, United States). Two separate multiplexed primer pools containing overlapping regions between, but not within the pools, were designed utilizing the Primal Scheme software^1^ in order to generate overlapping 400 base pair amplicons that spanned our entire viral genomes as seen in Supplementary Tables 1–3 (Quick et al., 2017). The amplicons were prepped for Illumina-based deep sequencing using the Nextera XT DNA Library preparation kit and the manufacturer’s instructions (Illumina, San Diego, CA, United States). The amplicon library was deep sequenced at the sequencing core facility at the University of Wisconsin, Madison, utilizing a MiSeq Illumina desktop sequencer (Invitrogen, Carlsbad, CA, United States). All sequence reads were prepared as FASTQ file format and analyzed as described below.

### Cell Culture

Whole blood filter packs were obtained from the Garth Englund Blood Center of Fort Collins, CO, United States. Mononuclear cells were isolated by Ficoll-Plaque density centrifugation. PBMC were grown and maintained in RPMI media containing 10% heat inactivated fetal bovine serum (HI FBS), 2× antibiotic-antimycotic mix (Thermo Fisher Scientific, Waltham, MA, United States) and 20 ng/mL IL-2 (R&D Systems, Inc., Minneapolis, MN, United States). For viral propagation, the PBMC were CD8 depleted by positive selection and then stimulated using 100 ng/mL of anti-CD3 and anti-CD28 soluble antibody (Miltenyi Biotec Inc., Auburn, CA, United States) for 48 h. The TZM-bl reporter and 293 cell lines were cultured in DMEM media containing 10% HI FBS, 1% antibiotic-antimycotic mix (Thermo Fisher Scientific, Waltham, MA, United States) and 1% L-glutamine.

### Viral Propagation, Titration and Subsequent Viral Passaging of SIVcpz

To propagate the virus from the first passage to the next, bone marrow, thymus, spleen, mesenteric and axillary lymph nodes, and whole blood obtained through cardiac puncture were harvested from the SIVcpz infected mice with the highest plasma viral titer. Lymphocyte fractions isolated by Ficoll-Plaque density centrifugation were seeded at a density of 2–3 × 10^6^ cells/mL and activated for 48 h with 100 ng/mL of anti-CD3 and anti-CD28 soluble antibody (Miltenyi Biotec Inc., Auburn, CA, United States). To assure viral infection of the next generation mice, these cells were then co-cultured for 48 h with fresh splenocytes obtained from the new hu-mice cohort used for serial passage. These cultured cells together with culture supernatants containing the virus were inoculated intraperitoneally to the next hu-mice batch of 5 mice.

### Quantification of Variant Frequencies

To calculate the variant frequencies in the sequencing datasets, a strategy similar to that we previously described in Schmitt et al. (2018). Briefly, we removed the primer sequences first by trimming 30 bases off the ends of the reads, then removed the adapter sequences and low-quality bases using the cutadapt software v1.9.1 (Martin, 2011). Filtered reads were mapped to stock virus consensus sequences using the bowtie2 software v2.2.5 (Langmead and Salzberg, 2012). Bowtie2 BAM format output was used as input to lofreq software v2.1.2 to call variants (Wilm et al., 2012). To qualify for a variant, we required >100 coverage and >1% frequency. The impact of variants was determined, and variants were plotted using R and ggplot2 (ISBN: 0387981403). Scripts are available at GitHub repositories^2^ ^,3^.

### Assessment of Amino Acid Frequencies in HIV-1 and SIV Database Sequences

To quantify the frequencies of specific amino acids in SIV and HIV sequences for use as a comparison available from the HIV Sequence Database Compendium^4^, multiple protein sequence alignments for each HIV-1/SIVcpz protein were downloaded from the 2017 compendium, which were the most recent alignments available for retrieval in April of 2019 (Foley et al., 2017). From the total HIV-1/SIVcpz alignments, subsets containing only HIV-1 or SIV sequences were extracted. These were used to create a position frequency matrix by tabulating, for each position (each column) of the alignment, the number of observations of each amino acid or gap character. A mapping of the amino acid position in the SIV protein to the position (column) in the multiple sequence alignment was then established for each protein of each of the three SIVcpz strains (EK505, MB897, and LB715). The position frequency matrix plus this mapping helped determine the frequency in these sets of HIV and SIV sequences of any variant that arose during the passage/generation experiments. The scripts and data files used to conduct this analysis are available at: https://github.com/stenglein-lab/SIV_to_HIV-1.

## Data Availability Statement

The raw data supporting the conclusions of this article is available on the sequence read archive (SRA) (BioSample accession numbers: SAMN15338262–SAMN15338315).

## Ethics Statement

The animal study was reviewed and approved by Colorado State University IACUC.

## Author Contributions

RA, KS, JC, MS, PM, and SO’C were responsible for the design and conduct of the project. LR-M, KG, and RM provided technical assistance. All authors contributed to the article and approved the submitted version.

## Conflict of Interest

The authors declare that the research was conducted in the absence of any commercial or financial relationships that could be construed as a potential conflict of interest.

## Footnotes

1 http://primal.zibraproject.org

2 https://github.com/stenglein-lab/SIV_to_HIV-1

3 https://github.com/stenglein-lab/viral_variant_explorer

4 http://www.hiv.lanl.gov/
