# Supplementary material for: Cross-Species Transmission and Evolution of SIV Chimpanzee Progenitor Viruses Toward HIV-1 in Humanized Mice
Source: Front Microbiol. 2020 Aug 11;11:1889. doi: 10.3389/fmicb.2020.01889 (PMC7432304; doi:10.3389/fmicb.2020.01889)
Supplement: Supplementary file 1 [file Data_Sheet_1.docx]

Ecosystem-Based Harvest Control Rules for Norwegian and US Ecosystems

## Supplementary Information

For “Ecosystem-Based Harvest Control Rules for Norwegian and US Ecosystems”

Isaac C. Kaplan, Cecilie Hansen, Hem Nalini Morzaria-Luna, Raphael Girardin , Kristin N. Marshall

**Table S1.** Median response to harvest control rules, across simulations of California Current (“Cal Cur.”) and Nordic and Barents Seas (“NOBA). This table summarizes fishery and ecological metrics in Figure 5 and comparable box plots. As in those box plots, responses are scored relative to comparable simulations with target fish F = F_MSY_.

|  |  | Focal fish biomass | Focal fish catch | Focal zooplankton | Pel bio/PP | Bio/PP | MTL bio | Predfish prop | Dem/ pel fish | Dem/pelagic | Dem bio/ PP | Dem catch | Fish catch | Value | Exp rate | Fish exp rate | MTL catch | Total catch | Pel catch | CV of focal fish catch |
| --- | --- | --- | --- | --- | --- | --- | --- | --- | --- | --- | --- | --- | --- | --- | --- | --- | --- | --- | --- | --- |
| Simple threshold | Cal Cur. | 1.49 | 1.10 | 1.00 | 1.00 | 1.00 | 1.00 | 1.04 | 0.99 | 1.00 | 1.00 | 1.00 | 1.02 | 1.00 | 0.96 | 1.01 | 1.00 | 1.01 | 1.01 | 1.30 |
|  | NOBA | 1.20 | 0.98 | 1.00 | 1.00 | 0.99 | 1.01 | 1.02 | 0.99 | 1.00 | 1.00 | 1.00 | 1.00 | 1.00 | 1.00 | 0.99 | 1.00 | 1.00 | 1.00 | 2.51 |
| Threshold, **decrease** fishing if zooplankton **< 50%** | Cal Cur. | 1.76 | 1.16 | 1.00 | 1.00 | 1.00 | 1.00 | 1.06 | 0.98 | 1.00 | 1.00 | 1.00 | 1.04 | 1.00 | 0.93 | 1.03 | 1.00 | 1.01 | 1.01 | 8.28 |
|  | NOBA | 1.94 | 0.81 | 1.00 | 0.99 | 0.96 | 1.03 | 1.07 | 0.95 | 1.00 | 1.00 | 1.00 | 0.96 | 0.7 | 0.96 | 0.92 | 1.00 | 0.97 | 0.97 | 99.27 |
| Threshold, **decrease** fishing if zooplankton **< 25%** | Cal Cur. | 1.67 | 1.12 | 1.00 | 1.00 | 1.00 | 1.00 | 1.06 | 0.99 | 1.00 | 1.00 | 1.00 | 1.02 | 1.00 | 0.94 | 1.01 | 1.00 | 1.01 | 1.01 | 7.15 |
|  | NOBA | 1.34 | 1.01 | 1.00 | 1.00 | 0.99 | 1.01 | 1.02 | 0.98 | 1.00 | 1.00 | 1.00 | 1.00 | 1.00 | 1.01 | 0.98 | 1.00 | 1.00 | 1.00 | 45.07 |
| Threshold, **increase** fishing if zooplankton **< 50%** | Cal Cur. | 1.42 | 1.12 | 1.00 | 1.00 | 1.00 | 1.00 | 1.03 | 0.99 | 1.00 | 1.00 | 1.00 | 1.02 | 1.00 | 0.96 | 1.02 | 1.00 | 1.01 | 1.01 | 1.79 |
|  | NOBA | 1.12 | 0.99 | 1.00 | 1.00 | 0.99 | 1.01 | 1.01 | 0.99 | 1.00 | 1.00 | 1.00 | 0.99 | 1.00 | 1.00 | 0.99 | 1.00 | 1.00 | 0.99 | 20.04 |
| Threshold, **increase** fishing if zooplankton **< 25%** | Cal Cur. | 1.44 | 1.09 | 1.00 | 1.00 | 1.00 | 1.00 | 1.04 | 0.99 | 1.00 | 1.00 | 1.00 | 1.02 | 1.00 | 0.97 | 1.02 | 1.00 | 1.01 | 1.01 | 1.85 |
|  | NOBA | 1.17 | 0.98 | 0.99 | 0.90 | 0.85 | 1.15 | 1.03 | 1.01 | 0.99 | 0.92 | 1.01 | 0.99 | 1.00 | 0.99 | 1.00 | 1.00 | 1.01 | 1.00 | 22.80 |

***
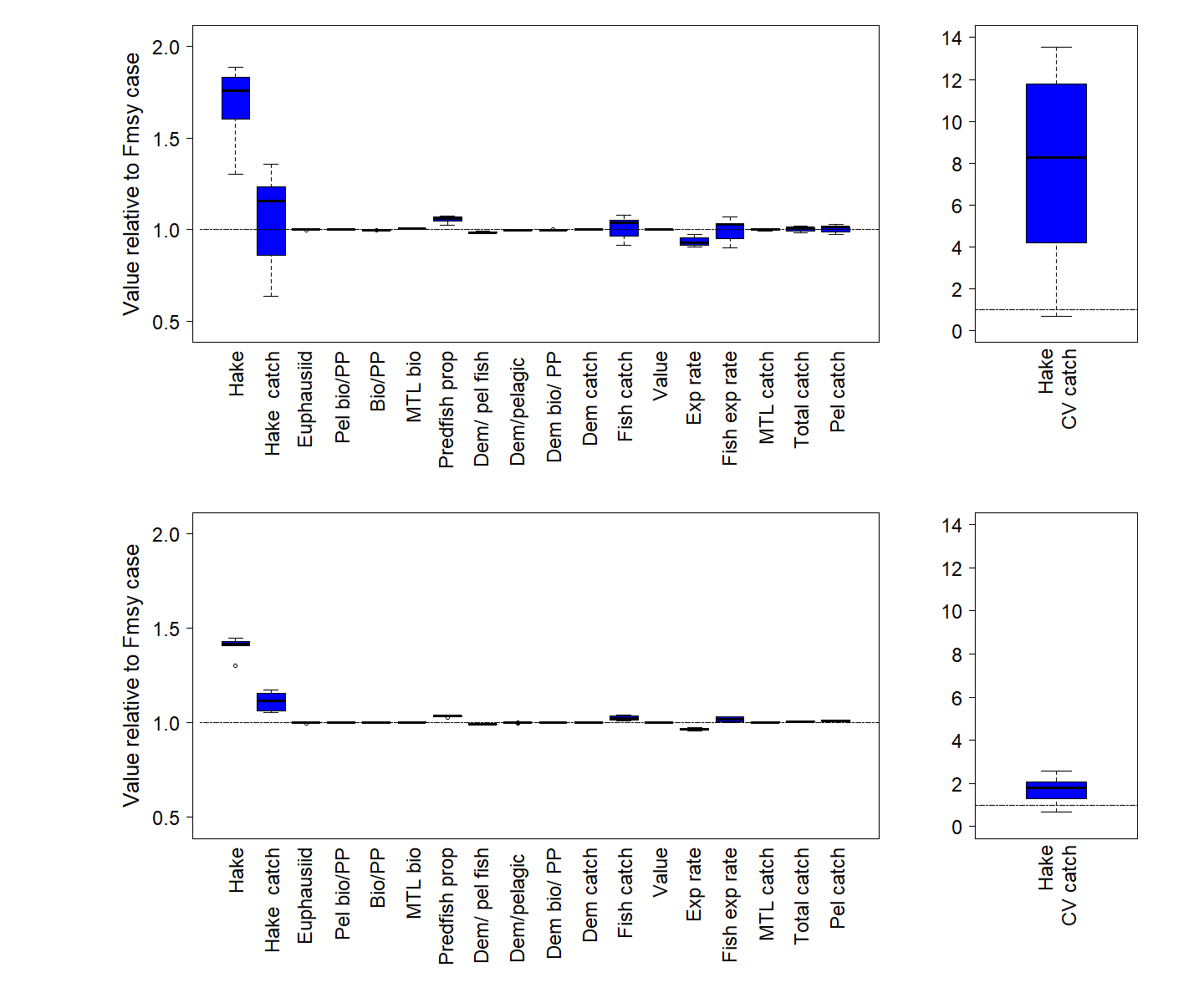
***

***Figure S1:*** *Fishery and ecological performance metrics for the California Current, similar to Figure 5. Top panel: Performance of a threshold rule for target fish that* ***decreases*** *fishing if productivity declines (if zooplankton <* ***50%****). This threshold rule is #3 in* ***Table 1.*** *Bottom panel: Performance of a threshold rule for target fish that* ***increases*** *fishing if productivity declines (if zooplankton <* ***50%****). This threshold rule is #5 in* ***Table 1.*** *Note y-axis limits for CV of catch differ from Figure 5.*

**
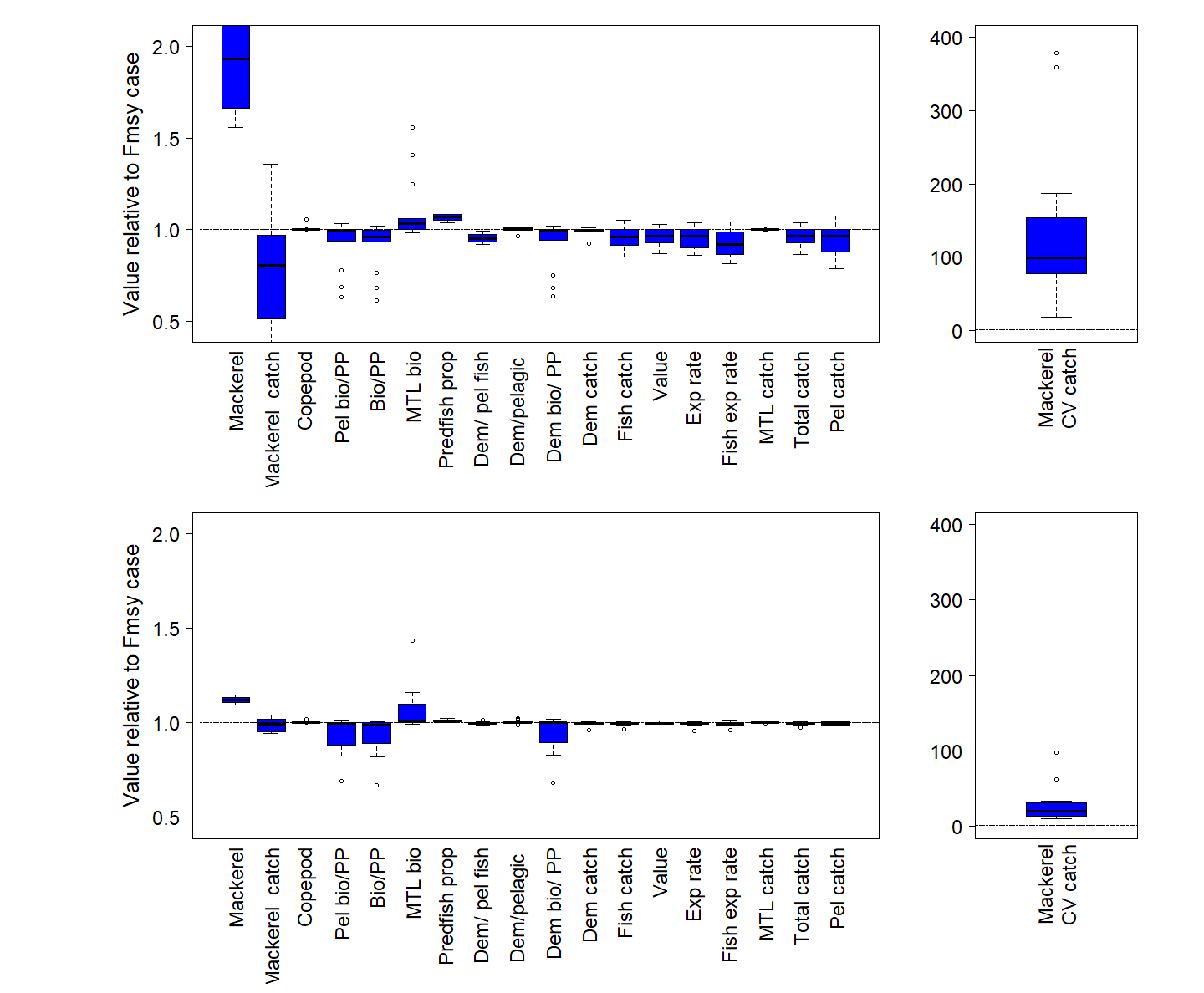
**

***Figure S2:*** *Fishery and ecological performance metrics for the Nordic and Barents Seas, similar to Figure 6. Top panel: Performance of a threshold rule for target fish that* ***decreases*** *fishing if productivity declines (if zooplankton <* ***50%****). This threshold rule is #3 in* ***Table 1.*** *Bottom panel: Performance of a threshold rule for target fish that* ***increases*** *fishing if productivity declines (if zooplankton <* ***50%****). This threshold rule is #5 in* ***Table 1.*** *Note y-axis limits for CV of catch differ from Figure 6.*

***
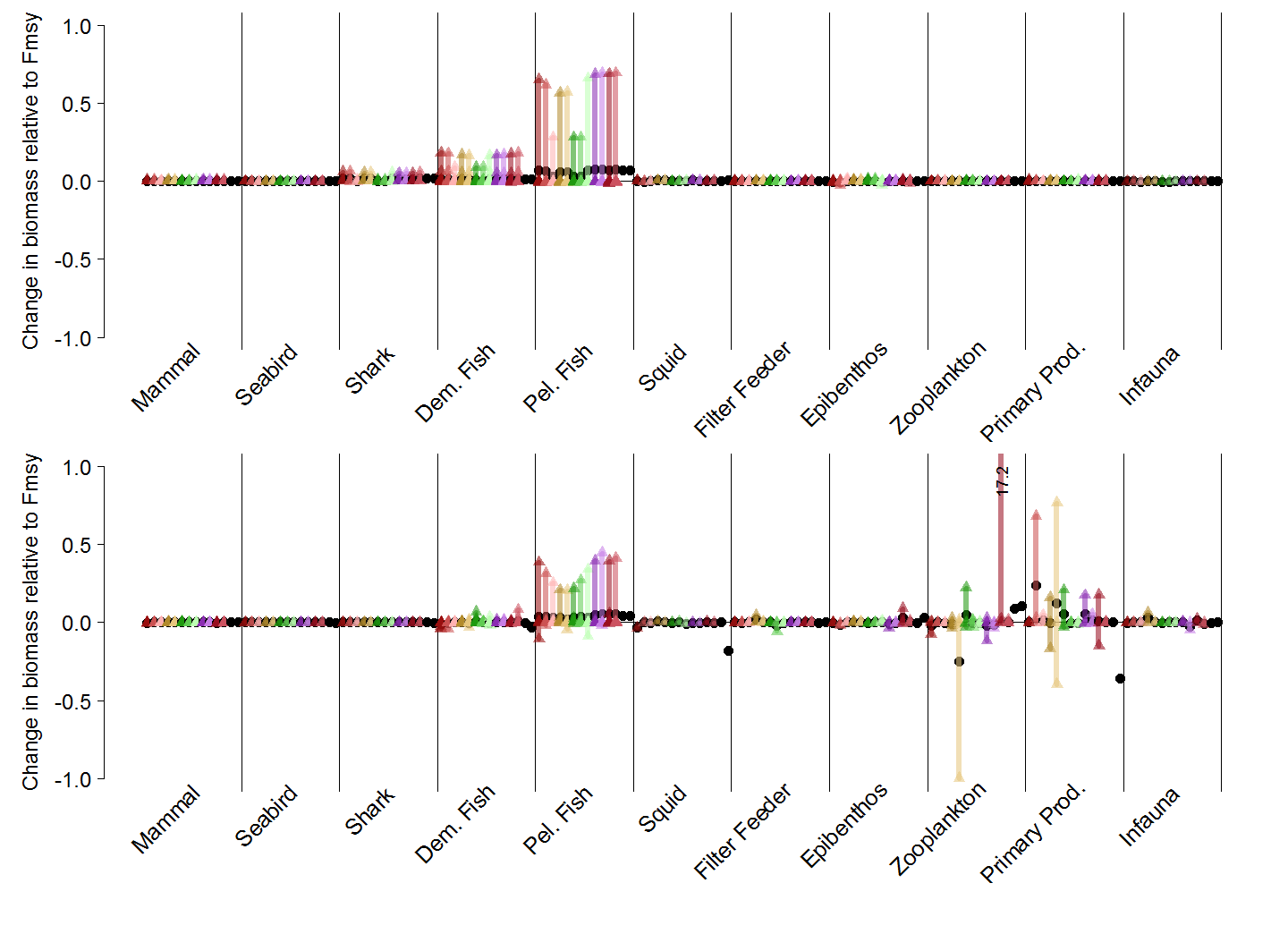
***

***Figure* S3.** *Guild-level biomasses for simulations with a threshold harvest control rule for target fish that* ***decreases*** *fishing if productivity declines (if zooplankton <25%). This threshold rule is #4 in* ***Table 1.*** *Biomasses are scored against comparable “control” simulations with target fish F= Fmsy. Each simulation is represented by a unique color. Vertical bars represent the range of functional group responses, grouped by guilds, within each simulation. Small triangles are individual functional group responses, and black circles are the average responses per simulation.* ***Top panel:*** *California Current results for Pacific hake as target fish.* ***Lower panel:*** *Nordic and Barents Seas results for mackerel as target fish.*

***
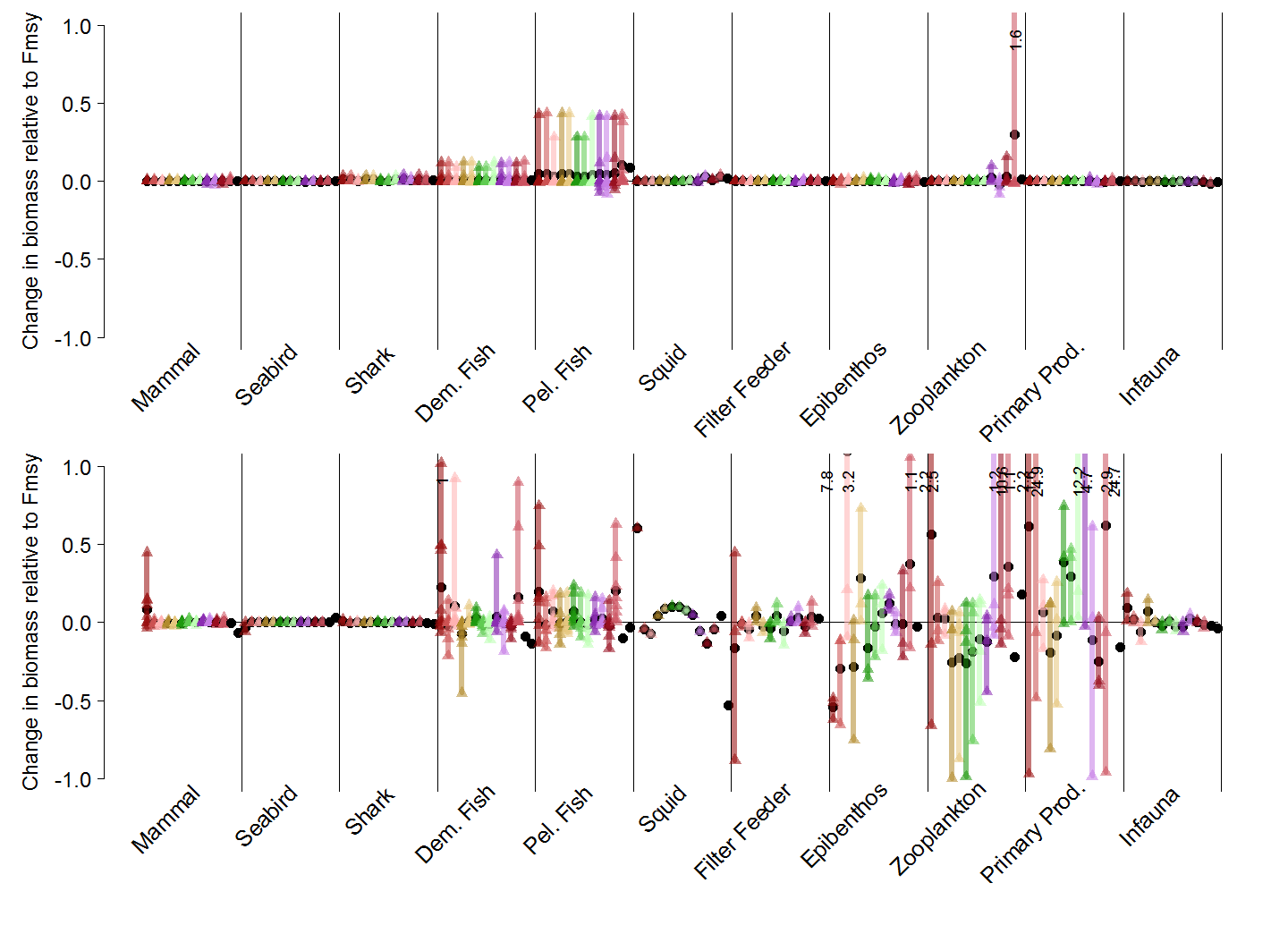
***

***Figure* S4*.*** *Guild-level biomasses for simulations with a threshold harvest control rule for target fish that* ***increases*** *fishing if productivity declines (if zooplankton <25%). This threshold rule is #6 in* ***Table 1.*** *Biomasses are scored against comparable “control” simulations with target fish F= Fmsy. Each simulation is represented by a unique color. Vertical bars represent the range of functional group responses, grouped by guilds, within each simulation. Small triangles are individual functional group responses, and black circles are the average responses per simulation.* ***Top panel:*** *California Current results for Pacific hake as target fish.* ***Lower panel:*** *Nordic and Barents Seas results for mackerel as target fish.*
